# Phenotypic and Physiological Evaluation of Two and Six Rows Barley under Different Environmental Conditions

**DOI:** 10.3390/plants7020039

**Published:** 2018-05-04

**Authors:** Mahmoud Naser, Mohamed Badran, Hanaa Abouzied, Heba Ali, Ibrahim Elbasyoni

**Affiliations:** 1 Crop Science Department, Faculty of Agriculture, Damanhour University, 22516 Damanhour, Egypt; m.abdelmoteleb@agr.dmu.edu.eg (M.N.); muhammad_badran@yahoo.com (M.B.); hmmahdy@yahoo.com (H.A.); 2 Barley Research Department, Field Crops Research Institute, Agricultural Research Center, 22571 Nubaria, Egypt; ebaali7599@yahoo.com

**Keywords:** imported barley, evaluation, genotypes, biotic stress, pre-breeding

## Abstract

In recent years, barley has attracted more interest as a food and feed source because of its high soluble dietary fiber and β-glucan content compared with other small grains. Twenty-five barley genotypes (20 imported genotypes and five check cultivars) were grown in three environments for two successive seasons: 2015/2016 and 2016/2017. The first environment was in El-Nubaria, Alexandria, Egypt during 2015/2016, while the second and third environments were in El-Bostan, Elbhera, Egypt during 2015/2016 and 2016/2017. The experiments were conducted in a randomized complete block design with the three replicates. The primary objectives of the current study were to evaluate the performance of 20 imported barley genotypes under several environmental conditions. The imported materials were superior to the local commercial cultivars for several traits, including grain yield. Therefore, the superior genotypes will be further evaluated and used in barley breeding programs. Our future work will focus on creating several crosses among the selected superior genotypes to improve yield and other important traits, while applying marker-assisted selection.

## 1. Introduction

Climate change and fluctuation in the environmental conditions imposed several abiotic and biotic stress conditions on field crops. Abiotic stress-tolerant field crops are the ultimate solution to cope with the projected climatic changes. Barley (*Hordeum vulgare* L.) proved to be one of the crops that can provide a source of abiotic stress tolerance genes, and thus grow under a wide range of environmental conditions [1]. Barley is the fourth most important cereal crop after wheat, rice, and maize [2]. Barley can be used as a nutritional source for both humans and livestock [3]. Moreover, barley grain contains about 20% of dietary fiber and β-glucan, which varies between 3–7% [4]. Martinez et al. [5] have shown that barley β-glucan has significant blood cholesterol-lowering effects. Barley β-glucan increases the viscosity of digestion in the intestine, slowing down the rate of starch digestion and absorption [6], which is beneficial to people with diabetes [7].

Barley has the potential to grow under limited water conditions and saline soils. Salinity-affected soils in Egypt account for about 33% of the total cultivated land [8]. Additionally, barley was found to be moderately tolerant to drought stress, which is useful because of the limited amount of water that is available for irrigation in Egypt [9]. Furthermore, rainfall in Egypt is less than 130 mm/year on average, while it exceeds 200 mm in some parts of the world; albeit, the productivity of rainfed barley in Egypt is higher than the average worldwide productivity [10]. Adverse environmental conditions in Egypt force barley breeders to import genotypes to cope with such low-input conditions. In addition to the limited water that is available for irrigation combined with the low precipitation in Egypt, there are other low-input conditions, such as poor soil fertility [10]. Overall, the harvested barley area was doubled during the last couple of years, in which it increased from 30.39 thousand hectares in 2000/2001 to 77.57 thousand hectares in 2015/2016. The total barley production increased from 93.9 thousand tons in 2000/2001 to 120.1 thousand ton in 2015/2016. Furthermore, barley production decreased from 3.03 tons/ha in the 2000/2001 growing season to 1.55 tons/ha in the 2015/2016 growing season [11]. The decline in barley productivity in Egypt might be attributed to adverse environmental conditions such as drought, salinity, reduced soil fertility, and high temperature, as well as certain biotic stresses such as leaf rust, powdery mildew, net blotch, and viral diseases. The lack of adequate genetic variability aggravated the adverse effect of previously mentioned biotic and abiotic stresses [10].

Therefore, it is necessary to increase the genetic variability of barley in Egypt in order to allow breeders to select new genotypes with high yield potential and more adaptability to climate change. On the other hand, the lack of phenotypic evaluation remains one of the serious constraints that limit the use of available plant collections across plant-breeding programs [12,13]. Therefore, pre-breeding became one of the essential steps to utilize plant genetic resources conserved in several comprehensive worldwide seed collections [13]. Pre-breeding is also one of the most effective approaches that can be used to improve cultivated genotypes using genes or traits from exotic materials [14]. The Global Crop Diversity Trust defined pre-breeding as “the art of identifying desired qualities and integrating them into modern breeding materials” [15]. Overall, pre-breeding refers to specific activities that are designed to identify desirable traits and genes from wild or exotic germplasm. Identifying such traits or genes is usually followed by transferring it into an intermediate set of plant materials that plant breeders could incorporate in his or her breeding efforts to develop improved genotypes [16,17].

The genetic base of the current cultivated barley materials in Egypt is limited [10], which raises a critical question about the resilience of the Egyptian barley-breeding programs to biotic outbreaks or anticipated fluctuations in the environmental conditions. In the current study, we evaluated imported barley genotypes under several environmental conditions side by side with locally adapted cultivars. Our future work will focus on crossing the superior exotic genotypes with local cultivars to form several families, giving that our ultimate goal is to increase yield production and maintain adequate genetic diversity for future improvements.

## 2. Materials and Methods

### 2.1. Plant Materials and Experimental Conditions

The present study was conducted in three environments (two locations in 2015/2016 and a single location in 2016/2017). The two locations were El-Nubaria, Alexandria, Egypt (30°53′36′′ N, 30°04′35′′ E), and El-Bostan Experimental Farm, Faculty of Agriculture, Damanhour University, Egypt (30°46′46′′ N, 30°82′32′′ E). During 2015/2016, the plant materials were planted in El-Bostan (E2) and El-Nubaria (E1). However, in 2016/2017, the studied materials were planted in El-Bostan only (E3).

The plant materials contained a collection of 20 barley lines that were obtained from the University of Minnesota, Minnesota, United States (USA) (Table 1) in addition to five check cultivars (“Giza123”, “Giza127”, “Giza132”, “Giza134”, and “Giza136”) obtained from the Agriculture Research Center (ARC), Egypt (Table 1). A randomized complete block design with three replicates was used in each environment. The plot size (experimental unit) was 3.75 m^2^ (4 rows ×1.5 m × 2.5 m). Seeding rate was 119 kg ha^−1^. Sowing dates were during the first half of November in the two growing seasons. Soil analysis was conducted on soil samples collected from 30-cm depth from each location according to Black et al. [18] (Supporting Information, Table S1). Additionally, the meteorological data are presented in the Supporting Information, Table S2. 

### 2.2. Studied Traits

The number of days to flowering (DF) was recorded when 50% of the spikes in a plot had extruded anthers (noted as days from 1 January). Plant height (PH) was measured on a random sample of five plants in each plot as the length from the soil surface to the tip of the spike at harvest time. A random sample of 10 spikes was collected from each plot, and the mean number of grains per spike (NG/S) for each plot was calculated. For the measurement of 1000-grain weight (TGW), 1000 seeds were taken randomly from each genotype grain yield and weighed. The grain yield (GY) was determined by harvesting the four rows of each plot, and expressed as tons/ha. Leaf chlorophyll content (CHL) was estimated using a spad-502 chlorophyll meter (Minolta, Japan). Leaf area (LA) was estimated according to [19] the equation as follows:

Leaf area (LA)=L×W×0.75

where L and W are the length and width of the flag leaf, respectively.

Leaf rust was visually scored as a percentage of leaf area infected for each barley plot under open field conditions. Leaf rust severity (%) was recorded for each genotype across the three environments using the modified Cobb’s scale [20]. Plant reaction (infection type) expressed in five types were: immune = O, resistant = R, moderately resistant = MR, moderately susceptible = MS, and susceptible = S [21]. The coefficient of infection for leaf rust has been calculated on the infection type after replacing the infection types with 0, 0.2, 0.4, 0.8, and 1 scores for immune, resistant, moderately resistant, moderately susceptible, and susceptible, respectively. Net blotch (*Pyrenophora teres* f. *teres*, NB) and powdery mildew (*Blumeria graminis* f. sp. *hordei*, PM) were visually scored as a percentage of leaf area infected for each plot under open field conditions. Plants with disease scores of 0 were classified as immune, plants that scored 1 and 2 were classified as resistant, plants that scored 3 and 4 were classified as moderately resistant, plants that scored 5 and 6 were classified as moderately susceptible, and plants with a score of 7 to 9 were classified as susceptible [22].

### 2.3. Statistical Analysis

Data were statistically analyzed using the analysis of variance procedures by SAS 9.2 (SAS v9.2; SAS Institute Inc., Cary, NC, USA) for a randomized complete block design as follows:

$$Y_{ij}=\mu +B_{j}+G_{i}+e_{ij}$$

where *Y_ij_* is the traits; *μ* is a population mean; *G_i_* is the effect of genotype *i*; *B_j_* is the effect of block *j*; and *e_ij_* is the experimental error.

The linear model for an across environments combined analysis of variance was conducted as follows:

$$Y_{ijk}=\mu +E_{i}+B_{k}\left(E_{i}\right)+G_{j}+{\left(GE\right)}_{ij}+e_{ijk}$$

where *μ* is a population mean; *E_i_* is the effect of environment *i*; *G_j_* is the effect of genotype *j*; (*GE*)*_ij_* is the interaction effect of *j* genotype × *i* environment; *B_k_* is the effect of block *k* in environmental *i*; and *e_ijk_* is the experimental error.

Means were compared using the *Lsd* test (*p*-value ≤ 0.05), according to Gomez et al. [23]. The homogeneity of variance in different environments was tested following Bartlett’s test [24]. Combined analyses of variance were performed among environments with homogeneous variance, as outlined by Cochran et al. [25]. Correlation coefficients were conducted using the Pearson correlation coefficient. A cluster analysis of the genotypes for all of the traits across the three environments was carried out using Ward’s method on the Euclidian distance [26]. A biplot for genotypes and traits was used to visualize the interaction between genotypes and environments using R software [27] and the FactoMineR package [28].

## 3. Results

The Bartlett’s test [24] results indicated homogeneous variance across environments for the number of days to flowering, plant height, number of grains/spike, leaf area, and grain yield. Whereas there was a non-homogeneous variance for 1000-grain weight, total chlorophyll content, leaf rust disease, net blotch disease, and powdery mildew disease. Therefore, a combined analysis of variance was conducted for only traits with homogeneous variance across the three environments.

### 3.1. Analysis of Variance

All of the morphological, physiological, and pathological traits that were measured (Table 2 and Table 3) revealed significant statistical differences among genotypes under the three environments except resistance to net blotch and powdery mildew diseases. There were non-significant differences among genotypes in E1 and E2 for net blotch resistance, while non-significant differences were observed among genotypes for powdery mildew resistance under E1 and E3.

The results of combined analysis of variance (Table 3) indicated highly significant (*p*-value ≤ 0.01) differences among the three environments for traits such as number of days to flowering, plant height, number of grains/spike, grain yield, and leaf area. The interaction between environment × genotypes was highly significant (*p*-value ≤ 0.01) for number of days to flowering, plant height, number of grains/spike, grain yield, and leaf area.

### 3.2. Traits Mean Across Genotypes and Environments

Mean values for the number of days to flowering (DF) was 66.24 days in E1, 70.4 days under E2, and 64.7 days in E3, with an overall mean of 67.1 days. The earliest genotype under the three environments was 07MN-82, with an average number of days of 56.2, while the latest genotype was 09WA-04, which flowered after 78.8 days (Supporting Information, Table S3). Furthermore, the mean of the plant height among the three environments was 89.8 cm in E1, 87.3 cm under E2, and 80.3 cm in E3, with an overall mean of 85.7 cm. The tallest genotype across three environments was 07N6-94 (96.1 cm), while the shortest genotype was 08N2-66, where it recorded 72 cm (Supporting Information, Table S3). The mean for the number of grains/spike (NG/S) among the three environments was 44.85 grains/spike in E1, and 47.09 grains/spike in E2, while it was 49 grains/spike in E3, with an overall mean of 46.98 grains/spike (Supporting Information, Table S4). Genotype 08UT-01 had the highest NG/S (78.1 grains/spike), but genotype 07N2-13 produced the lowest number of grains/spike (23.8 grains/spike) under the three environments.

Means of the 1000-grain weight (TGW) for the 25 genotypes under the three environments were 48.4 g, 34.9 g, and 37.2 g in E1, E2, and E3, respectively (Supporting Information, Table S4). In E1, the highest genotype in TGW was Giza 136 (68.3 g), while the lowest genotype was Giza 127 (28 g). In E2, the imported genotypes produced the highest and the lowest TGW, where the highest genotype was 08N2-18 (47 g), and the lowest genotypes were 08UT-85, 07UT-83, and 09UT-44, where they recorded 20 g. In E3, the highest genotype was 08N2-18 (52 g), while the lowest genotype was 07UT-83, where it recorded 24g. 

**Figure 1:**
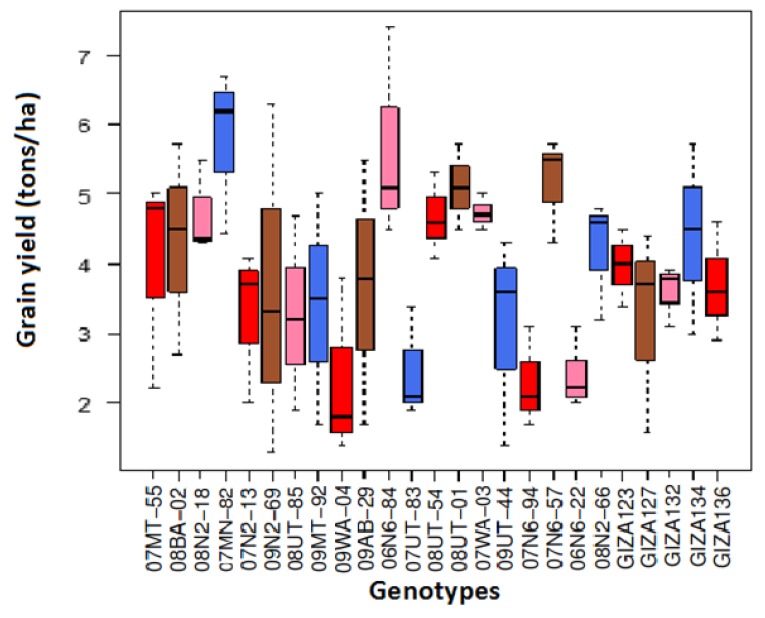
Boxplots for mean grain yield (GY) for 25 genotypes across the three environments.

The highest mean grain yield was 4.7 tons/ha in E3, while it was 3.7 tons/ha and 3.3 tons/ha in E1 and E2, respectively. The imported genotypes exceeded the Egyptian cultivars in grain yield (GY), where the highest imported genotype was 07MN-82 with 5.7 tons/ha. Moreover, the highest Egyptian cultivar was Giza 134 (4.4 tons/ha), and the lowest genotype in GY was 07N6-94, where it recorded 2.3 tons/ha (Figure 1 and Supporting Information, Table S4). 

The mean total chlorophyll content for the 25 genotypes across the three environments were 48.4 SPAD units, 45.96 SPAD units and 50.3 SPAD units under E1, E2 and E3, respectively (Supporting Information, Table S5). In E1, the highest genotype in total chlorophyll content was Giza 132 (74.3 SPAD units), while the lowest genotype was 09AB-29 (39.4 SPAD units). In E2, the highest genotype in regard to total chlorophyll content was 06N6-84 (72.7 SPAD units), while the lowest genotype was 07UT-83 (38 SPAD units). In the E3, the highest genotype was 07N2-13 (55.5 SPAD units), while the lowest genotype was 07UT-83 (37.7 SPAD units). Leaf area (LA) means were 15.83 cm^2^, 18.97 cm^2^, and 22.1 cm^2^ under E1, E2, and E3, respectively (Supporting Information, Table S5). The highest genotype for LA across the three environments was 09UT-44 (31.9 cm^2^), while the lowest genotype was 08N2-66 (9.7 cm^2^).

The mean coefficient values of infection for leaf rust (LR) across the three environments conditions were 25.6, 32 and 25 for E1, E2, and E3, respectively (Supporting Information, Table S6). In E1, the most resistant genotypes to leaf rust were Giza 134, followed by Giza 132, where they recorded 2, while the most susceptible genotypes were 08UT-85 and 09N2-69, where they recorded 63.3.

**Figure 2:**
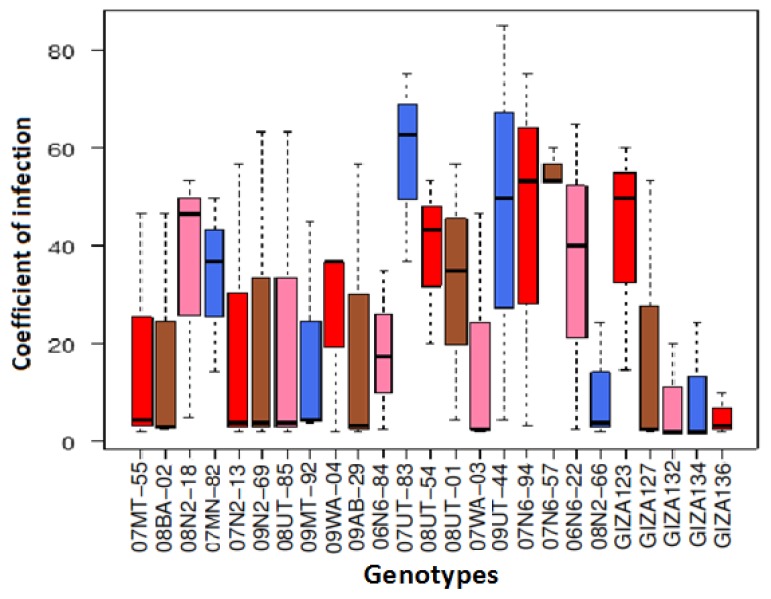
Boxplots for the mean coefficient of infection for leaf rust (LR) for 25 genotypes across the three environments.

In E2, the most resistant genotypes to leaf rust disease were 07N2-13, 09N2-69, 09AB-29, 08UT-85, 09MT-92, and 08N2-66, where they recorded 4, while the most susceptible genotype to leaf rust was 07UT-83, where it recorded 63 (Supporting Information, Table S6). In E3, the most resistant genotypes were 07N2-13, 09N2-69, 09WA-04, 09AB-29, 08UT-85, 07WA-03, 08N2-66, Giza132, Giza134, Giza127, and Giza136, where they recorded 2, while the most susceptible genotype was 09UT-44, where it recorded 85 (Supporting Information, Table S6). Overall, the most resistant genotype under the three environments to leaf rust disease was Giza 136, where it recorded 5.1, while the most susceptible genotype was 07UT-83, where it recorded 58.2 (Figure 2).

The mean values of the net blotch resistance scores (NB) were 1.2, 1, and 2 for E1, E2, and E3, respectively. In E3, among the 20 imported genotypes tested, 14 genotypes (08BA-02, 07MN-82, 09N2-69, 08UT-85, 09WA-04, 09AB-29, 06N6-84, 07UT-83, 08UT-01, 09UT-44, 07N6-94, 07N6-57, 06N6-22, and 08N2-66) exhibited a high resistance response to NB disease, where they recorded 1, while two genotypes (08N2-18 and 09MT-92) exhibited a moderately resistant response for NB disease, where they recorded 3 and 4, respectively. Two genotypes—08UT-54 and 07WA-03—exhibited a moderate susceptibility response for NB disease, where they recorded 5.6 and 5.3, respectively. Furthermore, two genotypes (07N2-13 and 07MT-55) exhibited a susceptible response for NB disease (6.6 and 6.5 respectively). Additionally, the Egyptian cultivars (Giza123, Giza 127, Giza 132, Giza 134, and Giza 136) exhibited a high resistance response for NB disease, where they recorded 1 (Supporting Information, Table S6). 

On the other hand, the mean values of the powdery mildew resistance scores (PM) were 1, 3.5, and 1 for E1, E2, and E3, respectively (Supporting Information, Table S6). In E2, among the 20 imported genotypes tested, seven genotypes (08BA-02, 08N2-18, 07MN-82, 09N2-69, 09WA-04, 06N6-84, and 06N6-22) exhibited a high resistance response to PM, where they recorded 2, 2.6, 2.6, 2.6, 1.6, 2.6, and 2.6, respectively. Five genotypes (07MT-55, 08UT-85, 07WA-03, 07N6-57, and 08N2-66) exhibited a moderately resistant response for PM, where they recorded 3. Another five genotypes (07N2-13, 09MT-92, 09AB-29, 08UT-01, and 07N6-94) exhibited a moderately susceptible response for PM disease, where they recorded 5, 4.3, 5, 5.6, and 5, respectively.

Three genotypes (07UT-83, 08UT-54, and 09UT-44) exhibited a susceptible response for PM disease, where they recorded 6, 6, and 8, respectively. The Egyptian cultivars (Giza134 and Giza127) exhibited a high resistance response for PM, where they recorded 0.66 and 1.6, respectively. Cultivars Giza132, Giza136, and Giza123 exhibited a moderately resistant response for PM, where they recorded 2.6, 2, and 3, respectively (Supporting Information, Table S6). 

### 3.3. Relationships among the Studied Traits

**Figure 3:**
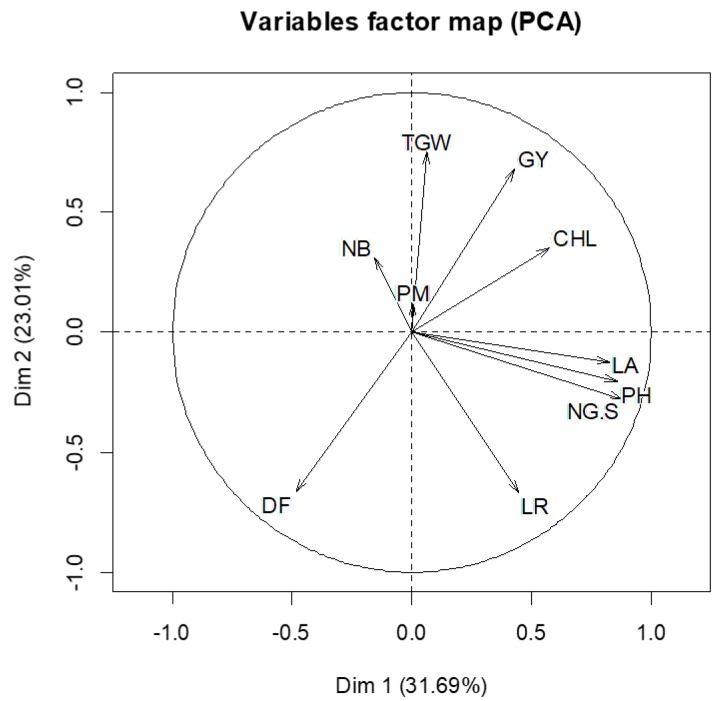
Biplot showing the correlation among total chlorophyll content (CHL), number of days to flowering (DF), plant height (PH), number of grains/spike(NG/S),1000-grain weight (TGW), leaf rust (LR), net blotch (NB), powdery mildew (PM), grain yield (GY), and leaf area (LA).

The first two principal components accounted for 31.69% and 23.01% of the total variability (Figure 3). The correlation coefficients among all of the traits under the three environments are shown in Table 4. The results revealed a significant positive correlation (*p*-value ≤ 0.05) among total chlorophyll content, leaf area, and plant height. Moreover, our results indicate non-significant correlation (*p*-value > 0.05) between total chlorophyll content and grain yield, number of grains/spike, 1000-grain weight, leaf rust, net blotch, and powdery mildew. Meanwhile, the correlation was significant and negative among the number of days to flowering, 1000-grain weight, and grain yield (Table 4). Furthermore, the correlation between the number of days to flowering and total chlorophyll content was significant and negative. Our results also indicate non-significant correlation for the number of days to flowering with plant height, number of grains/spike, leaf rust, net blotch, powdery mildew, and leaf area. Correlations of plant height with the number of grains/spike, leaf rust, and leaf area were significant and positive. Meanwhile, the correlation of plant height with 1000-grain weight, net blotch, powdery mildew, and grain yield were non-significant. The number of grains/spike was positive and significantly correlated with leaf rust and leaf area, but it was non-significantly correlated with 1000-grain weight, net blotch, powdery mildew, and grain yield. 1000-grain weight was significantly and positively correlated with grain yield, while it was non-significantly correlated with leaf rust, net blotch, powdery mildew, and leaf area. Leaf rust was significantly and positively correlated with leaf area, but it was not significantly correlated with net blotch, powdery mildew, and grain yield. The correlation between net blotch and powdery mildew was positive and significant, while it was not significantly correlated with leaf area and grain yield. Powdery mildew was not significantly correlated with leaf area and grain yield. Finally, the correlation between leaf area and grain yield was not significant. 

### 3.4. Relationships among Barley Genotypes 

**Figure 4:**
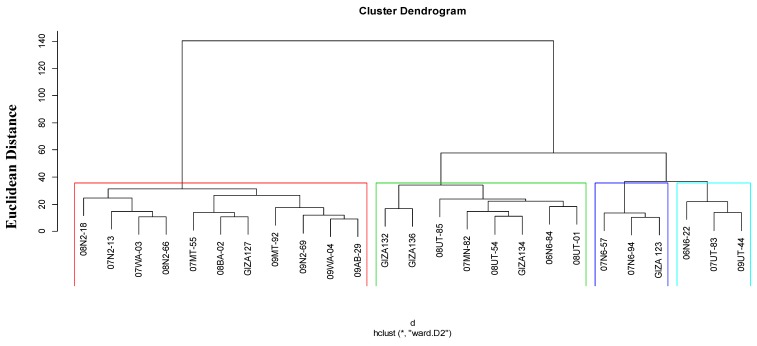
Dendrogram of cluster analysis of 25 barley genotypes based on morphological, physiological, and pathological traits across the three environments.

A cluster analysis for the 25 barley genotypes based on the phenotypic, physiological, and pathological traits using Ward’s method is depicted in Figure 4. Across the three environments, conditions cluster analysis assigned the genotypes into four clusters. The first cluster consisted of 11 genotypes (08N2-18, 07MT-55, 08BA-02, 07N2-13, 09N2-69, 09MT-92, 09WA-04, 09AB-29, 07WA-03, 08N2-66, and Giza127) and represented 44% of the total genotypes; this group represented mainly the two-row genotypes. The second group contained 32% of the total genotypes (08UT-85, 07MN-82, 08UT-54, 06N6-84, 08UT-01, Giza132, Giza134, and Giza136). Meanwhile, genotypes 07N6-94, Giza123, and 07N6-57 were classified in the third cluster, which represented 12% of the total genotypes. Furthermore, the fourth cluster contained 06N6-22, 07UT-83, and 09UT-44, and accounted for 12% of the total genotypes.

## 4. Discussion

Twenty barley genotypes were imported from the University of Minnesota, which were evaluated under the field conditions in Egypt. Our results indicated superior performance for the imported genotypes in grain yield, number of days to flowering, plant height, number of grains/spike, 1000-grain weight, and leaf area. Our ultimate objective was to identify potential genotypes suitable to the Egyptian growth conditions (Supporting Information, Table S2). The imported barley genotypes outperformed the Egyptian cultivars in traits such as grain yield, number of grains/spike, 1000-grain weight, and leaf area, which agrees with previous reports [10,25]. Meanwhile, the Egyptian cultivars outperformed the imported genotypes for total chlorophyll content and resistance to leaf rust, net blotch, and powdery mildew diseases. These results could be due to the higher adaptability in the local cultivars compared with the imported materials [29,30]. The three environments used in this study were selected on the basis of their differences in agro-ecological, soil texture, and pathological features. The different response that was observed among genotypes within the same environment may be attributed to the different origin and genetic background [31]. As a result, the cluster analysis assigned the genotypes into four clusters that were similar in morphological, physiological, and pathological traits. On the other hand, genotype × environment interaction was observed among genotypes in which genotypes responded differently across the three environments. These results were in agreement with previous studies [32,33,34] where the variation in the environmental and soil conditions was found to contribute to differences in morphological traits such as grain yield, number of grains/spike, leaf area, chlorophyll content, plant height, and grain weight for the same genotypes. Additionally, the third environment (E3) was more suitable for most of the barley genotypes due to exposing the genotypes to favorable growth conditions, which resulted in the better overall performance of the genotypes under this environment [35,36]. The results of barley resistance to leaf rust, net blotch, and powdery mildew diseases indicated a correlation between these diseases and the eco-geographical characteristics of the locations used, including average annual rainfall, altitude, and average annual temperature [37,38]. Our results highlighted the importance of testing genotypes under different environmental conditions to identify the best genotypes for a particular environment [29]. The 25 genotypes used in this study were evaluated for morphological traits, grain yield, some yield components, and physiological and pathological traits. The importance of these traits comes from using them to identify the most adapted genotypes. Also, these traits are essential and useful for plant breeders seeking to increase genetic variability and yield production [30,31,39,40,41]. Furthermore, our results indicated that the number of days to flowering, plant height, number of grains/spike, leaf area, and grain yield were less affected by the environmental conditions, which agree with previous reports [40,42,43], and might explain the homogeneity of variance across the environments for those traits. The combined analysis of variance for homogeneous traits indicated that the genotypic variance explained 57.9%, 38%, 91%, 41.9%, and 54,6% of the total variance for traits such as the number of days to flowering, plant height, number of grains/spike, grain yield, and leaf area, respectively. The obtained results were in agreement with previous reports in which the kernel weight, grain yield, number of days to flowering, leaf area, number of kernels per main spike, biological yield per plant, and plant height were found to have high to moderate genotypic variance [3,40,43,44,45]; therefore, selection can be based on these characters. The determination of correlation coefficients among traits helps obtain the best combinations of attributes for obtaining a higher yield per unit area [46]. In the current study, the correlation between grain yield and number of days to flowering was negative and significant. These results are compatible with Ismail and Khokhar et al. [47,48]. On the other hand, the correlation between grain yield and 1000-grain weight was positive and significant, which agrees with previously reported results [49,50]. Our overall results indicated the superiority of some of the imported barley genotypes (07MN-82, 06N6-84, 08UT-01, 07N6-57, 08N2-18, 07WA-03 and 08UT-54) compared with the local commercial cultivars for grain yield and other important traits.

## 5. Conclusions 

The results reported indicate the possibility of cultivating these genotypes directly in Egypt and exploiting their genetic backgrounds via crosses with the local cultivars. We expect that crossing the selected barley materials with the local cultivars might enhance the genetic variability of the Egyptian barley, while improving yield potentials. Our future work will focus on creating several crosses among the selected superior genotypes to improve yield and other important traits, while applying marker-assisted selection. 

## Figures and Tables

**Table 1:** Identification and pedigree of the studied barley genotypes: 20 imported genotypes from the United States (USA), and five Egyptian cultivars.

NO	Genotype	Synonym	Row Type	Pedigree	Origin
1	MT070107	07MT-55	2R	MT970148/Haxby	USA
2	Z155U012V	08BA-02	2R	Z010C020E/Z011L088L	
3	2ND27344	08N2-18	2R	ND24289/ND22996//CONLON	
4	NEG2-03	07MN-82	6R	M119/M99-106 NB	
5	2ND26318	07N2-13	2R	ND21957-2/ND23024	
6	2ND27678	09N2-69	2R	2ND24175/TR05285	
7	2159-90	08UT-85	6R	AB 11469 × Aquila	
8	MT090234	09MT-92	2R	MT910189/MT910189/Lk644/Eslick BC3F3 7-L	
9	07WA-605.17	09WA-04	2R	WA 9004-99/Merit	
10	2AB08-X05M046-21	09AB-29	2R	2AB08-X05M046-21	
11	ND23366	06N6-84	6R	ND19656/ND19703	
12	2142-69	07UT-83	6R	SDB11009/M72395/3/Short2//ID633019/Woodvale/4/Steptoe/M27//Gusto/5/WA11825-95/6//Harrington	
13	2155-54	08UT-54	6R	Goldeneye × UT97B1556-5344	
14	2152-3	08UT-01	6R	Morex × Goldeneye	
15	05WA-319.12	07WA-03	2R	Bob/Merit//CDC Select	
16	UT2170-146	09UT-44	6R	Harrington/3/Kombar/SF8623//Maranna	
17	ND25736	07N6-94	6R	ND19474/ND20477	
18	ND25697	07N6-57	6R	STELLAR-ND/ND20603	
19	ND23541	06N6-22	6R	Drummond/ND20414	
20	2ND27392	08N2-66	2R	ND24519/CONLON	
21	Giza123	-	6R	GIZA117/FAO86	Egypt
22	Giza127	-	2R	W12291/Bags//Harmal-02	
23	Giza132	-	6R	Rihane-05//AS 46/Aths*2Athe/Lignee 686	
24	Giza134	-	6R	-	
25	Giza136	-	6R	-	

*: Refer to backcrossing.

**Table 2:** Analysis of variance for 1000-grain weight (g), leaf chlorophyll content, leaf rust, net blotch and powdery mildew of 25 barley genotypes that across E1 (El-Nubaria station 2015/2016), E2 (El-Bostan 2015/2016) and E3 (El-Bostan 2016/2017).

Trait	Source of Variance	df	Mean Squares		
			E1	E2	E3
1000-grain weight	Genotypes	24	326.8 **	179.52 **	6.99 **
	Replicates	2	2.89	17.33	0.64
Leaf chlorophyll content	Genotypes	24	249.5 **	136.46 **	54.61 **
	Replicates	2	10.68	124.4	9.488
Leaf rust	Genotypes	24	25.60 **	20.694 **	2321.01 **
	Replicates	2	0.093	0.28	7.413
Net blotch	Genotypes	24	1 ^ns^	1 ^ns^	11.32 **
	Replicates	2	1	1	0.56
Powdery mildew	Genotypes	24	1 ^ns^	12.97 **	1 ^ns^
	Replicates	2	1	0.77	1

^ns^: non-significant and **: Significant at *p*-value = 0.01.

**Table 3:** Combined analysis of variance for the number of days to flowering, plant height (cm), number of grains/spike, grain yield (tons/ha), and leaf area (cm^2^) of 25 barley genotypes (G) across three environments (E).

Trait	Source of Variance	df	S.S	MS
Number of days to flowering	E	2	1281.7	640.87 **
	Replicates (E)	6	16.68	2.78
	G	24	9618.4	400.76 **
	E × G	48	3846	80.12 **
Plant height	E	2	4567.1	2283.5 **
	Replicates (E)	6	85.55	14.26
	G	24	13,262.9	552.6 **
	E × G	48	10,347.7	215.58 **
Number of grains/spike	E	2	675	337.5 **
	Replicates (E)	6	123.2	20.5
	G	24	66796	2783.2 **
	E × G	48	3764	78.42 **
Grain yield	E	2	86.28	43.14 **
	Replicates (E)	6	3.01	0.50
	G	24	250.43	10.43 **
	E × G	48	177.65	3.70 **
Leaf area	E	2	1508.8	754.4 **
	Replicates (E)	6	91.85	15.31
	G	24	8292.6	345.5 **
	E × G	48	2335.3	48.7 **

**: Significant at 0.01.

**Table 4:** Correlation coefficients among the studied traits across the three environments.

	CHL	DF	PH	NG/S	TGW	LR	NB	PM	LA	GY
**CHL**	1									
**DF**	−0.4 *	1								
**PH**	0.41 *	−0.33	1							
**NG/S**	0.25	−0.26	0.76 **	1						
**TGW**	0.23	−0.42 *	−0.10	−0.17	1					
**LR**	−0.001	0.20	0.43 *	0.50 *	−0.33	1				
**NB**	0.12	−0.15	−0.33	−0.22	−0.07	−0.14	1			
**PM**	0.02	−0.11	−0.19	0.02	−0.17	0.02	0.88 **	1		
**LA**	0.43 *	−0.18	0.56 *	0.77 **	−0.07	0.44 *	0.07	0.29	1	
**GY**	0.27	−0.55 **	0.21	0.22	0.54 **	−0.25	−0.01	−0.1	0.26	1

* and **: Significant at *p*-value = 0.05 and 0.01. CHL: total chlorophyll content, DF: number of days to flowering, PH: plant height, NG/S: number of grains/spike, TGW: 1000-grain weight, LR: leaf rust, NB: net blotch, PM: powdery mildew, GY: grain yield, and LA: leaf area.

## Supplementary Materials

The following are available online at http://www.mdpi.com/2223-7747/7/2/39/s1, Table S1: Physical and chemical soil properties for E1 (El-Nubaria station 2015/2016), E2 (El-Bostan 2015/2016) and E3 (El-Bostan 2016/2017). Table S2: Meteorological parameters for E1 (El-Nubaria station 2015/2016), E2 (El-Bostan 2015/2016) and E3 (El-Bostan 2016/2017). Table S3: Genotypes means for the number of days to flowering (days) and plant height (cm), under three environments, E1 (El-Nubaria station 2015/2016), E2 (El-Bostan 2015/2016) and E3 (El-Bostan 2016/2017). Table S4: Genotypes means for number of grains/spike, 1000- grain weight (g) and grain yield (tons/ha), across E1 (El-Nubaria station 2015/2016), E2 (El-Bostan 2015/2016) and E3 (El-Bostan 2016/2017). Table S5: Genotypes means for total chlorophyll (Spad unit) and leaf area (cm^2^), under three environments, E1 (El-Nubaria station 2015/2016), E2 (El-Bostan 2015/2016) and E3 (El-Bostan 2016/2017). Table S6: Genotypes means for leaf rust, net blotch, and powdery mildew, across E1 (El-Nubaria station 2015/2016), E2 (El-Bostan 2015/2016) and E3 (El-Bostan 2016/2017).
