# Supplementary material for: Phenotypic and Physiological Evaluation of Two and Six Rows Barley under Different Environmental Conditions
Source: Plants (Basel). 2018 May 4;7(2):39. doi: 10.3390/plants7020039 (PMC6027403; doi:10.3390/plants7020039)
Supplement: Supplementary file 1 [file plants-07-00039-s001.pdf]

Table S1. Physical and chemical soil properties for E1 (El-Nubaria station 2015/2016), E2 (El-Bostan 2015/2016) and E3 (El-Bostan 2016/2017).

Environments / Physical properties	E1	E2	E3
Clay%	31.84	0.9	1.1
Silt%	19.9	1.5	1.4
Sand%	48.26	97.6	97.5
Soil texture	Sandy clay loam	Sand	Sand
Chemical properties	value	value	value
pH	8.11	7.7	8.5
EC(dsm^{-1})	3.2	1.85	0.71
$CaCO_3$	25.93	6.1	4.16
Organic matter %	0.55	0.04	0.05
Soluble cations meq100-1 g soil	value	value	value
Ca^{++}	5.4	6.1	1.64
Mg^{++}	4.2	3.0	1.67
Na^{++}	16.3	9.5	3.49
K^{+}	3.8	0.1	1.87
Soluble anions $meq100^{-1}$ g soil			
HCO_3	9.8	1.8	2.1
Cl^{-}	17.2	9.8	3.6
SO_4	2.7	7.1	1.48

Table S2. Meteorological parameters for E1 (El-Nubaria station 2015/2016), E2 (El-Bostan 2015/2016) and E3 (El-Bostan 2016/2017).

Parameters / Month	Air Temp (°c)						Relative Humidity (%)			Wind Speed (Km/h)		
	E1		E2		E3		E1	E2	E3	E1	E2	E3
	Min	Max	Min	Max	Min	Max						
November	19.0	24.0	20.0	25.0	15.1	25.5	61.30	25.4	25.2	13.0	13.0	12.0
December	13.0	19.0	18.0	20.0	13.9	16.8	65.25	26.5	28.8	11.0	12.0	13.0
January	10.0	17.0	16.9	18.6	13.5	17.7	85.7	28.5	24.3	13.0	13.6	13.5
February	11.0	21.0	17.4	21.5	14.5	18.4	64.4	27.2	22	12.0	12.0	13.0
March	13.0	23.0	19.2	23.1	15.5	21.1	59.3	24.8	21.4	13.0	13.0	12.0
April	16.0	27.0	21.25	28.5	17	28.3	58.5	23.7	22.1	11.0	11.1	11.0
May	19.0	28.0	23.3	29.20	17.4	29.3	54	22.6	22.7	13.0	12.6	13.0
Mean	14.4	22.7	19.4	23.7	15.23	22.4	64.1	25.5	23.8	12.3	12.5	12.5

* Source: Central Laboratory for Agricultural Climate (CLAC), Cairo, Egypt.

Table S3. Genotypes means for the number of days to flowering (days) and plant height (cm), under three environments, E1 (El-Nubaria station 2015/2016), E2 (El-Bostan 2015/2016) and E3 (El-Bostan 2016/2017).

	No. of days to flowering				Plant height			
Genotypes	E1	E2	E3	Mean	E1	E2	E3	Mean
07MT-55	62	73.6	68	67.8	93	95	75	87.6
08BA-02	74	74	67	71.6	83	90.6	75	82.8
08N2-18	60	66.6	60.6	62.4	76.6	94.6	86	85.7
07MN-82	52.6	58	57	56.2	91.3	81.6	90	87.6
07N2-13	66	63.3	57	62.1	75	83.6	67.6	75.4
09N2-69	66	81.5	64.3	70.6	77.3	85.3	76. 7	79.7
08UT-85	64	74	68	68.6	97.3	93	82.3	90.8
09MT-92	81.5	83.3	67	77.3	73	81.6	80	78.2
09WA-04	80	87	69.6	78.8	73	78.3	75	75.4
GIZA134	62	62.6	59. 7	71.4	100	98.3	85	94.4
09AB-29	81.6	82.3	71.7	78.5	72.6	80.3	83	78.7
06N6-84	64	73	59	65.3	100.6	95.6	84.3	93.5
07UT-83	66	84	79	76.3	109.6	83	81.3	91.3
GIZA132	63.6	61	61.6	62.1	110	101	75	95.3

Table S3. Cont.

08UT-54	62	64	59. 7	61.8	110	92	90	94.3
08UT-01	62	69	64	65	96.6	94.3	87.3	92.7
GIZA127	68.6	65.3	62.66	65.5	76.6	93.3	89	86.3
07WA-03	77.3	65.3	65.66	69.4	73.3	86.6	66	75.3
09UT-44	74	85.6	68.33	76	95.6	92.6	88	92
07N6-94	54	63.5	68	61.7	109	93	86.3	96.1
07N6-57	62	58.6	59. 7	60.1	96.3	87.3	100	94.5
GIZA136	62	60	59	60.3	103.6	44.6	75	74.4
06N6-22	64.6	80.6	78.333	74.5	92	87.3	57	78.7
08N2-66	60	62.3	66	62.7	75	68.5	72.67	72
GIZA123	66	61	57	61.3	93	99.3	80	90.7
Mean	66.24	70.4	64.7	67.11	89.8	87.3	80.3	85.7
$LSD_{0.05}$	5.02	8.3	3.1	4.33^{++}	6.291	24.081	11.397	8.96^{++}

- $^{++}$To compare genotypes mean across three environments.

Table S4. Genotypes means for number of grains/spike, 1000- grain weight (g) and grain yield (tons/ha), across E1 (El-Nubaria station 2015/2016), E2 (El-Bostan 2015/2016) and E3 (El-Bostan 2016/2017)

	Number of grains/spike				1000- grain weight				Grain yield			
Genotypes	E1	E2	E3	Mean	E1	E2	E3	Mean	E1	E2	E3	Mean
07MT-55	31.1	25.3	29	28.5	44	30	38	37.3	2.2	4.8	5	3.9
08BA-02	27	28.3	32	29.1	34	36.6	38	36.2	2.7	4.5	5.7	4.3
08N2-18	27.6	27.6	29	28.1	66	47	52	54.8	4.3	4.36	5.5	4.7
07MN-82	60.3	71.5	78	69.6	60	40	32	44	6.7	4.42	6.2	5.7
07N2-13	26.6	23	22	23.8	44	30	40	38	3.7	2	4.1	3.2
09N2-69	26.9	30	31	29.2	50	30	46	42	3.3	1.3	6.3	3.6
08UT-85	57	66.7	71.6	65.1	37.3	20	27	27.7	3.2	1.9	4.7	3.3
09MT-92	35.6	27	33	31.8	42	30	36	36	3.5	1.7	5	3.4
09WA-04	27	27	32	28.6	38	40	38	38.6	1.8	1.4	3.8	2.4
GIZA134	64	60	57	60.3	50	36.6	42	42.8	3	4.5	5.7	4.4
09AB-29	29	29.6	27.6	28.7	48	46.6	40	44.8	3.8	1.7	5.5	3.6
06N6-84	62	66.6	63	63.8	55	33.3	30	34.4	7.4	4.5	5.1	5.6
07UT-83	47	69	65.6	60.5	35	20	24	26.3	3.4	2.1	1.9	2.4
GIZA132	54.6	63.6	54	57.4	64.3	36.6	34	45	3.1	3.8	3.9	3.6

Table S4. Cont.

08UT-54	67	64.6	63	64.8	51.6	46.6	34	44.1	4.1	4.6	5.3	4.6
08UT-01	79.6	73.8	81	78.1	55	35	34	41.3	5.1	4.5	5.7	5.1
GIZA127	22.6	29	30	27.2	28	30	32	30	1.6	3.7	4.4	3.3
07WA-03	32	28	30	30	56	43.3	42	47.1	4.5	5	4.7	4.7
09UT-44	65.3	64.2	60.6	63.4	37.6	20	34	30.5	3.6	1.4	4.3	3.1
07N6-94	47	63.7	60	56.9	45	35	40	40	3.1	1.7	2.1	2.3
07N6-57	58.3	52.3	68	59.5	50	36.6	38	41.5	5.5	4.3	5.7	5.1
GIZA136	45.3	45.5	57	49.2	68.3	40	42	50.1	2.9	3.6	4.6	3.7
06N6-22	54	63.5	57.3	58.2	41.6	30	36	35.8	3.1	2	2.2	2.4
08N2-66	27	27.3	29.3	27.8	54	40	44	46	3.2	4.8	4.6	4.2
GIZA123	47	49.6	66	54.2	54.6	30	40	41.5	3.4	4a	4.5	3.99
Mean	44.85	47.09	49	46.98	48.4	34.9	37.2	40.1	3.7	3.3	4.7	3.9
$LSD_{0.05}$	1.97	6.53	7.6	4.37^{++}	4.12	9.07	0.811	ns	0.92	1.25	1.15	1.01^{++}

- ns: non- significant.
- $^{++}$To compare genotypes mean under the three environments.

Table S5. Genotypes means for total chlorophyll (Spad unit) and leaf area (cm^2), under three environments, E1 (El-Nubaria station 2015/2016), E2 (El-Bostan 2015/2016) and E3 (El-Bostan 2016/2017).

	Total chlorophyll				leaf area			
Genotypes	E1	E2	E3	Mean	E1	E2	E3	Mean
07MT-55	64.6	41.5	48.9	51.6	12	6.9	13.8	10.9
08BA-02	40.2	38.5	52.5	43.74	8.7	11.4	12.6	10.9
08N2-18	51.1	51.3	46.6	49.6	17	25.3	20.5	20.9
07MN-82	45e	47.5	53.8	48.7	23.5	19.6	27.4	23.5
07N2-13	44.8	47.8	55.5	49.3	13.8	10.2	15.3	13.1
09N2-69	47	46.1	48.6	47.23	17.7	17.8	20.8	18.8
08UT-85	40.6	39.7	55.2	45.17	15.5	21	25.9	20.8
09MT-92	43.9	40.7	48.6	44.4	11.5	15.9	14.6	13.8
09WA-04	47.5	39.7	54.3	47.16	13.1	11.2	16.3	13.5
GIZA134	46.3	42.3	50.8	46.5	17.1	20.7	21	19.6
09AB-29	39.4	44.9	44.3	42.86	14.1	14.7	19.03	15.9
06N6-84	41.8	72.7	52.1	55.5	21.4	29.3	30.4	28.7
07UT-83	68.5	38	37.7	48.1	18.2	12.6	33.1	21.3
GIZA132	74.3	48.2	52.6	58.4	16.2	30.6	22.2	23
08UT-54	56.1	43.8	44.5f	48.16	14.9	27.6	22.7	21

Table S5. Cont.

08UT-01	48.6	47.4	51.7	49.2	17.5	28.5	30.1	25.4
GIZA127	45.3	42.7	53.3	47.1	8.6	11.1	12.6	10.9
07WA-03	44.3	47.9	46.8	46.3	7.8	12.3	12.5	10.8
09UT-44	58	45.5	54.9	53	26.5	36.7	37.7	31.9
07N6-94	45.1	50.5	50.2	48.6	16.7	14	19.4	16.7
07N6-57	42.6	49	51.5	47.7	21.7	28.1	33.4	27.8
GIZA136	40.9	48.7	54.9	48.18	25.3	22.4	22.9	23.6
06N6-22	41.6	47.1	46.3	45.02	21.1	19.1	25.7	22
08N2-66	46.1	42	48.7	45.61	6	5.8	17.4	9.7
GIZA123	46	44.8	53.3	48.05	9	20.9	26.3	18.6
Mean	48.4	45.96	50.31	48.2	15.83	18.97	22.1	18.9
$LSD_{0.05}$	12.25	14.69	6.07	ns	7.147	8.07	7.05	6.4^{++}

- ns: non -significant.
- $^{++}$To compare genotypes mean across the three environments.

Table S6. Genotypes means for leaf rust, net blotch, and powdery mildew, across E1 (El-Nubaria station 2015/2016), E2 (El-Bostan 2015/2016) and E3 (El-Bostan 2016/2017).

Genotypes	**Leaf rust**				**Net blotch**				**Powdery mildew**			
	E1	E2	E3	Mean	E1	E2	E3	Mean	E1	E2	E3	Mean
07MT-55	4.6	46.6	26	25.7	1	1	6.5	2.8	1	3	1	1.6
08BA-02	2.6	46.6	3	17.4	1	1	1	1	1	2	1	1.3
08N2-18	53.3	46.6	5	34.9	1	1	3	1.6	1	2.6	1	1.5
07MN-82	50	36.6	14.3	33.6	1	1	1	1	1	2.6	1	1.5
07N2-13	56.6	4	2	20.8	1	1	6.6	2.88	1	5	1	2.3
09N2-69	63.3	4	2	23.1	1	1	1	1	1	2.6	1	1.5
08UT-85	63.3	4	2	23.1	1	1	1	1	1	3	1	1.6
09MT-92	4.6	4	45	17.86	1	1	4	2	1	4.3	1	2.1
09WA-04	36.6	36.6	2	25	1	1	1	1	1	1.6	1	1.2
GIZA134	2	24.6	2	9.5	1	1	1	1	1	0.66	1	0.9
09AB-29	56.6	4	2	20.6	1	1	1	1	1	5	1	2.3
06N6-84	2.6	17.3	35	18.3	1	1	1	1	1	2.6	1	1.5
07UT-83	36.6	63	75	58.2	1	1	1	1	1	6	1	2.7
GIZA132	2	20	2	8	1	1	1	1	1	2.6	1	1.5

Table S6. Cont.

08UT-54	43.3	53.3	20	38.86	1	1	5.6	2.5	1	6	1	2.7
08UT-01	4.6	56.6	35	32.06	1	1	1	1	1	5.6	1	2.5
GIZA127	2.6	53.3	2	19.3	1	1	1	1	1	1.6	1	1.1
07WA-03	2.6	46.6	2	17.06	1	1	5.3	2.4	1	3	1	1.6
09UT-44	50	4.6	85	46.5	7	7	1	3	1	8	1	3
07N6-94	3.3	53.3	75	43.9	1	1	1	1	1	5	1	2.3
07N6-57	53.3	60	53.3	55.5	1	1	1	1	1	3	1	1.6
GIZA136	3.3	10	2	5.1	1	1	1	1	1	2	1	1.3
06N6-22	2.6	40	65	35.9	1	1	1	1	1	2.6	1	1.5
08N2-66	24.6	4	2	10.2	1	1	1	1	1	3	1	1.6
GIZA123	14.6	60	50	41.5	1	1	1	1	1	3	1	1.6
Mean	25.6	32	25	27.5	1.2	1	2	1.4	1	3.5	1	1.83
$LSD_{0.05}$	13.89	13.95	7.19	ns	ns	ns	1.9	ns	ns	1.3	ns	ns

- ns: non- significant.
